# Time-Resolved Transcriptomic Profiling of Chandipura Virus Infection Reveals Dynamic Host Responses and Host-Directed Therapeutic Targets

**DOI:** 10.3390/ijms27083364

**Published:** 2026-04-09

**Authors:** Dhwani Jhala, Prachi Shah, Dhruvi Shah, Ishan Raval, Apurvasinh Puvar, Snehal Bagatharia, Naveen Kumar, Chaitanya Joshi, Amrutlal K. Patel

**Affiliations:** 1 Gujarat Biotechnology Research Centre, Gandhinagar 382011, India; 2 National Institute of Virology, Pune 411001, India

**Keywords:** Chandipura virus, RNA-seq, host–virus interaction, host-directed antivirals, drug screening, in vitro validation

## Abstract

Chandipura virus (CHPV) is a neurotropic rhabdovirus associated with recurrent outbreaks of acute encephalitis in children and a high case fatality rate, particularly in India. Despite its public health relevance, the host molecular processes governing CHPV infection and disease progression remain poorly defined. To address this gap, we conducted a time-resolved transcriptomic analysis to characterize host responses to CHPV infection and to explore host-directed therapeutic opportunities. Human HEK293T cells were infected with CHPV, followed by RNA sequencing (RNA-seq) at 6, 12, 18, and 24 h post infection (hpi). Transcriptome profiling revealed a temporally ordered host response. At 6 hpi, CHPV infection was dominated by strong activation of innate immune and inflammatory pathways, including interferon-stimulated genes and cytokine signaling. Antiviral responses persisted at 12 hpi, accompanied by suppression of metabolic and translational processes, indicating a shift in host cellular priorities. By 18 hpi, metabolic reprogramming—particularly involving lipid and sphingolipid metabolism—was observed alongside altered immune signaling, consistent with viral exploitation of host cellular machinery. At 24 hpi, repression of genes involved in chromatin organization, RNA processing, spliceosome assembly, and ribosome biogenesis reflected a global transcriptional shutdown associated with cytopathic effects. Integration of temporal transcriptomic signatures enabled identification of host pathways amenable to pharmacological targeting. Selected host-directed compounds were evaluated in vitro and exhibited antiviral activity against CHPV in a neuronal cell line. Collectively, this study provides the first time-resolved transcriptomic landscape of CHPV infection in human cells and identifies host-targeted strategies relevant for antiviral development.

## 1. Introduction

Chandipura virus (CHPV) is a pathogenic neurotropic virus known to cause acute encephalitis, predominantly in children. It belongs to the genus *Vesiculovirus* within the family *Rhabdoviridae*. CHPV closely resembles Vesicular Stomatitis Virus but has an additional ability to infect humans, unlike VSV. The virus was first identified from the blood of two adults suffering from febrile illness in the village of Chandipura, Maharashtra, India, from which it derives its name [1]. Since its discovery, CHPV has been recognized as an emerging human pathogen responsible for multiple encephalitis outbreaks, primarily in India, but also reported in Nigeria and Sri Lanka [2,3]. The virus is endemic in several Indian states, with outbreaks typically occurring during the monsoon season. One of the largest outbreaks occurred in 2003, resulting in 329 suspected cases and 183 deaths [4]. The most recent major outbreak, reported in Gujarat in 2024, recorded 245 cases and 82 deaths up to August 2024, as per World Health Organization (WHO) records [5,6].

CHPV is a negative-sense, single-stranded RNA virus with an approximately 11 kb genome (NCBI ID: PQ185534.2). Its genome encodes five major structural proteins—nucleoprotein (N), phosphoprotein (P), matrix protein (M), glycoprotein (G), and the large RNA-dependent RNA polymerase (L)—which together govern viral replication, assembly, and immune evasion [7]. The virus is primarily transmitted through sandflies (*Phlebotomus papatasi*), although mosquitoes and ticks have also been implicated as potential vectors [8]. Serological evidence suggests exposure in a wide range of vertebrate hosts, and the virus can replicate efficiently in both insect and vertebrate cell lines [9,10]. Children under 15 years of age are particularly susceptible to CHPV infection, which can lead to acute neurological manifestations. Clinical symptoms range from fever, vomiting, and convulsions to coma and acute encephalitis, with reported case fatality rates reaching up to 75% [11]. As with other RNA viruses, CHPV exhibits a high mutation rate, which facilitates rapid adaptation and contributes to its epidemic potential [12]. Despite its clinical significance, the molecular mechanisms underlying CHPV–host interactions remain poorly characterized. Most existing studies have focused on viral isolation, vector transmission, and epidemiology, with limited investigation into how CHPV reprograms host cellular pathways during infection.

Transcriptomic analysis represents a crucial aspect of the genomic landscape, capturing molecular changes associated with disease pathogenesis and treatment [13]. Transcriptomic analysis of host cellular responses to viral infection can be utilized to investigate potential cellular factors that are related to viral infection. RNA sequencing (RNA-seq) offers a comprehensive approach to unravel host–virus interactions by capturing genome-wide transcriptional changes in infected cells. Previous reports on viral infections such as SARS-CoV-2 [14,15,16], H5N1 influenza virus [17], Fowl adenovirus [18], Gemykibivirus [19], and Monkeypox virus [20] demonstrate the usefulness of transcriptomic approach in identifying host genes and pathways altered during infection. In addition, several studies, particularly on SARS-CoV-2, have utilized omics-based analyses to uncover virus–host interactions and identify potential therapeutic targets [21,22,23]. However, comprehensive transcriptomic analyses investigating host responses to Chandipura virus infection remain limited.

This study aims to define the temporal transcriptomic landscape of CHPV infection in HEK293T cells at multiple time points after infection. By integrating differential gene expression with gene ontology and KEGG pathway enrichment analyses, we systematically characterize the dynamic changes in host immune, metabolic, and regulatory networks throughout infection. This study represents the first comprehensive time-resolved transcriptomic profiling of CHPV infection and provides crucial insights into the molecular mechanisms of CHPV pathogenesis. Further, from the transcriptomic analysis, we used computational drug repurposing methods by publicly available gene-disease drug analysis. A few computational approaches, such as molecular docking/simulation and quantitative structure-activity relationship methods, have already been used for the identification of repurposable drugs [24]. Drug target identification is one of the most critical steps in the drug development pipeline. By utilizing increasingly large human genomics and proteomics datasets, computational approaches facilitate in silico target discovery while reducing evaluation time and associated costs [25]. Network-based approaches are advanced computational models for target identification that integrate concepts from network pharmacology, network medicine, systems biology, and multi-omics analysis, enabling biomarker discovery and the identification of diagnostic and therapeutic targets [26]. According to the published data, there are no antiviral medications available for CHPV and the development of a novel therapeutic agent is a prolonged and highly expensive process, often spanning several years and requiring substantial financial investment. To overcome this issue, the repurposing of USFDA approved drugs is a promising idea which is rapid, safer, and economically preferable. To experimentally validate the findings from the transcriptomic analysis, in vitro testing of drugs such as sulfasalazine, romidepsin, and nilotinib was performed. These drugs were administered in cultured human neuroblastoma SHSY5Y cells to evaluate their cellular effects.

## 2. Results

### 2.1. Infection of Chandipura Virus in HEK293T Cells

**Figure 1:**
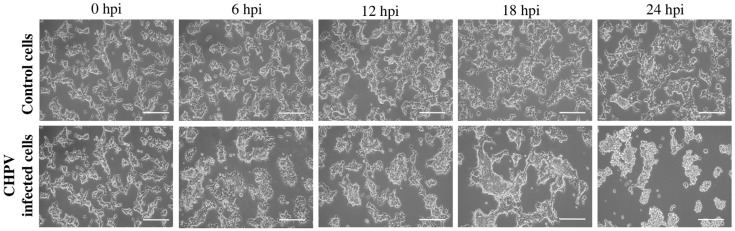
Microscopic images of HEK293T cells infected with Chandipura virus at different time points. Representative bright-field images of HEK293T cells at 0, 6, 12, 18, and 24 h after infection (hpi). The top row shows uninfected control cells at corresponding time points. The bottom row shows infected cells, demonstrating time-dependent cytopathic effects, including cell rounding and detachment. Scale bar: 200 μm.

In order to find the optimized dilution of CHPV virus for accurate and timely observation of viral pathogenesis in cells, we optimized different dilutions of CHPV virus in HEK293T cells (Figure S1a). The progression of infection was monitored through observation of cytopathic effects (CPE) at different time points. The aim was to select a dilution, at which cells survive up to 24 h, so that time-based changes in the transcriptome can be properly analyzed. Based on this, 10^−4^ dilution was selected for infecting the HEK293T cells in triplicates. The infected cells showed changes in their morphology as early as 6 h after infection, starting from rounding of cells to detachment from the surface by 22–24 h (Figure 1).

### 2.2. RNA Isolation, Library Preparation, and Sequencing

Total RNA was isolated from the harvested cells. RNA concentration measured by Qubit fluorometer (Thermo Fisher Scientific, San Jose, CA, USA) showed adequate yield. For quality analysis, RNA was run on agarose gel electrophoresis (Figure S1b). The gel image demonstrated distinct and sharp 28S and 18S ribosomal RNA bands at all time points, indicating high-quality, intact RNA suitable for downstream transcriptomic analyses. No significant degradation or smearing was observed, which confirmed the integrity of RNA samples. The transcriptome libraries were prepared and analyzed using a LabChip nucleic acid analyzer (Revvity, Waltham, MA, USA). The average size of the library was found to be 350 bp. The libraries were pooled at a final concentration of 4 nM and sequenced using the NovaSeq 6000 platform (Illumina, San Diego, CA, USA).

### 2.3. Evaluation of the RNA-Seq Data and Read Assembly

After RNA sequencing, the obtained reads were passed through QC and trimming and a total of 608.21 million clean reads were obtained, with an average of 40.54 million reads per sample. The Q30 percentage of the dataset exceeded 95%, and the GC content for all samples ranged between 44.01% and 47.7% (Table S1). The reads were then mapped using the STAR alignment tool, in which 84.62% of the reads mapped to the reference human genome GRCh38.p14 (hg38, *Homo sapiens*).

### 2.4. Identification of Differentially Expressed Genes at Different Time Points

**Figure 2:**
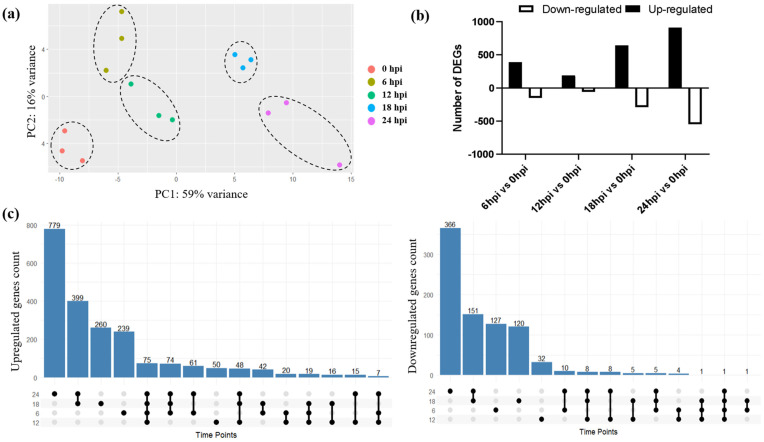
Transcriptome analysis of differentially expressed genes (DEGs) across time points after infection. (**a**) Principal Component Analysis (PCA) plot showing clustering of samples (represented by dotted lines) based on gene expression profiles at each time point (0, 6, 12, 18, and 24 hpi), indicating time-dependent transcriptomic shifts. (**b**) Bar plots depicting the number of significantly upregulated and downregulated genes (adjusted *p*-value < 0.05, |log_2_FC| ± 1) for each time point compared to 0 hpi. (**c**) Upset plots showing the number of unique and overlapping upregulated (**left**) and downregulated (**right**) DEGs across different time points.

The gene count data for each sample was used to perform differential expression analysis using the DESeq2 package (Supplementary File). Principal component analysis (PCA) of the differentially expressed genes (DEGs) revealed distinct separation of samples at different time points (0, 6, 12, 18 and 24 hpi), where triplicate samples at each time point clustered together (Figure 2a). Using the criteria of adjusted *p*-value ≤ 0.05 and |log_2_ fold change| ≥ ±1, a total of 3178 DEGs were identified across all time points (Figure 2b).

Among these, at 6 hpi vs. 0 hpi, 388 genes were upregulated and 149 were downregulated; at 12 hpi vs. 0 hpi, 191 genes were upregulated and 59 were downregulated. Similarly, at 18 hpi vs. 0 hpi, 641 genes were upregulated and 292 were downregulated, and at 24 hpi vs. 0 hpi, 909 genes were upregulated while 549 were downregulated. Additionally, 76 DEGs were commonly upregulated or downregulated across all time points, whereas 366, 82, 380, and 1145 DEGs were found to be unique to 6 hpi, 12 hpi, 18 hpi, and 24 hpi, respectively (Figure 2c). It is notable here that at 12 hpi, the total number of deregulated genes were low compared to other time points across the triplicates. The relatively lower number of differentially expressed genes at 12 hpi likely reflects a transitional phase of CHPV infection, during which early innate immune responses initiated at 6 hpi are partially stabilized before the onset of extensive metabolic reprogramming and host transcriptional shutdown observed at later time points.

**Figure 3:**
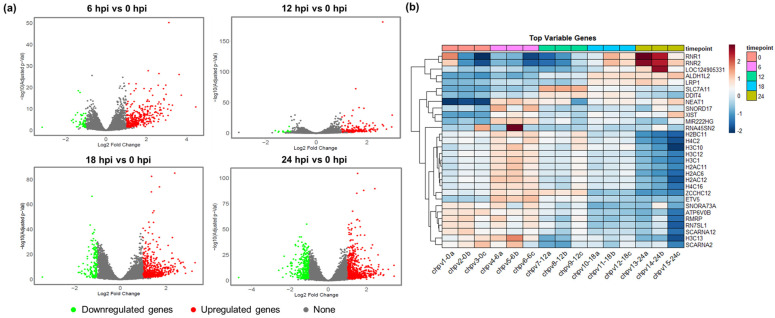
Differential expression profiles of host genes in response to Chandipura virus infection. (**a**) Volcano plots showing the distribution of DEGs (red: upregulated, green: downregulated) at 6, 12, 18 and 24 hpi versus control (0 hpi). Colored dots represent significant genes (adjusted *p*-value < 0.05, |log_2_FC| ± 1). (**b**) Heatmap showing the top 30 most variable genes across samples. Gene expression counts were normalized using variance stabilizing transformation (DESeq2) and mean-centered per gene. Columns represent biological replicates at each time point (0, 6, 12, 18, and 24 hpi). Red indicates higher expression and blue indicates lower expression relative to the gene’s average across samples. Genes were hierarchically clustered.

The volcano plots for DEGs at each time point further show the temporal dynamics of the host response (Figure 3a). At 6 and 12 hpi, a modest number of genes were upregulated, indicating early activation of innate immunity. By 18 and 24 hpi, both upregulated and downregulated genes significantly increased, indicating a complex interplay of immune activation and host gene suppression. Hierarchical clustering of the top 30 most variable genes across samples revealed distinct temporal expression patterns during infection (Figure 3b). Several genes (e.g., RNR1, ALDH1L2) exhibited elevated expression at later stages of infection (18–24 hpi), whereas others (e.g., SNORA73A, H3C13) showed relatively higher expression at earlier time points (0–6 hpi). Additionally, hierarchical clustering of all DEGs showed clear segregation of samples by infection time point, with biological replicates clustering together, demonstrating high reproducibility and consistent temporal transcriptional responses (Figure S1c). To visually examine the transcriptional dynamics of selected immune response genes (ISG15, IRF1, TRIM56, CCL27) during CHPV infection, RNA-seq coverage tracks were generated and inspected across different time points. Visualization of normalized coverage revealed temporal variation in read density across several immune-related loci during infection (Figure S2).

### 2.5. Biological Process and KEGG Pathway Enrichment at Different Time Points

To understand host–pathogen interactions and functional implications of CHPV-induced gene expression changes, we performed Gene Ontology (GO) biological process and KEGG pathway enrichment analyses at all time points. Table S2 enlists DEGs annotated with biological processes for 6, 12, 18 and 24 hpi vs. 0 hpi comparisons.

**Figure 4:**
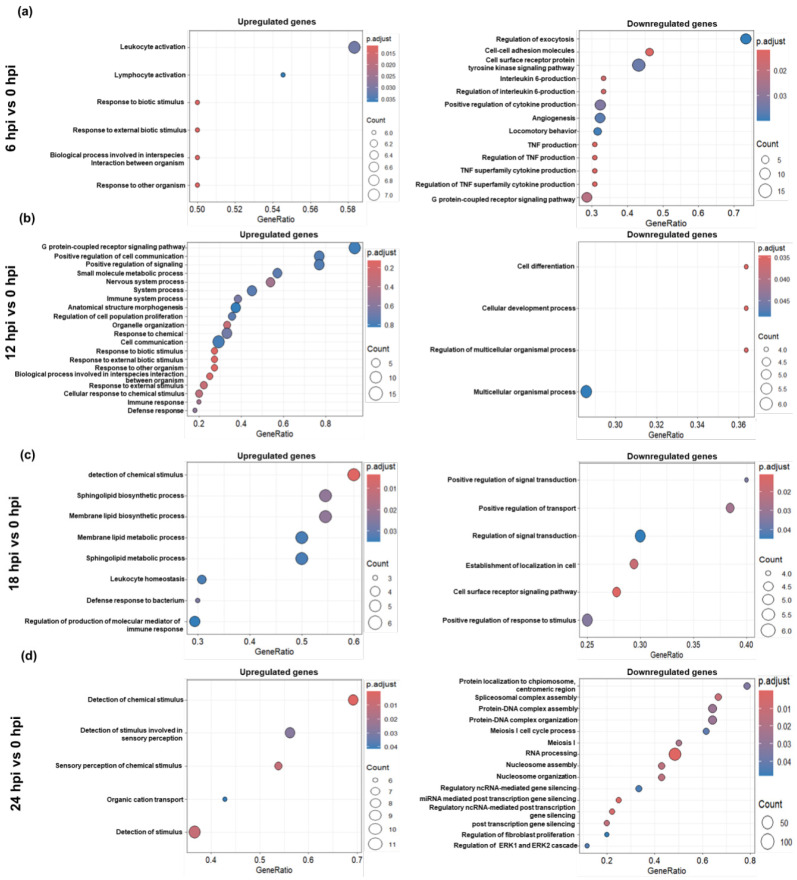
Biological pathway enrichment of differentially expressed genes across time points. Dot plots representing enriched Gene Ontology (GO) biological processes for DEGs at 6 hpi vs. 0 hpi (**a**), 12 hpi vs. 0 hpi (**b**), 18 hpi vs. 0 hpi (**c**), and 24 hpi vs. 0 hpi (**d**). The size of the dots indicates the number of genes associated with each term, while color intensity corresponds to the statistical significance (−log10 adjusted *p*-value).

**Figure 5:**
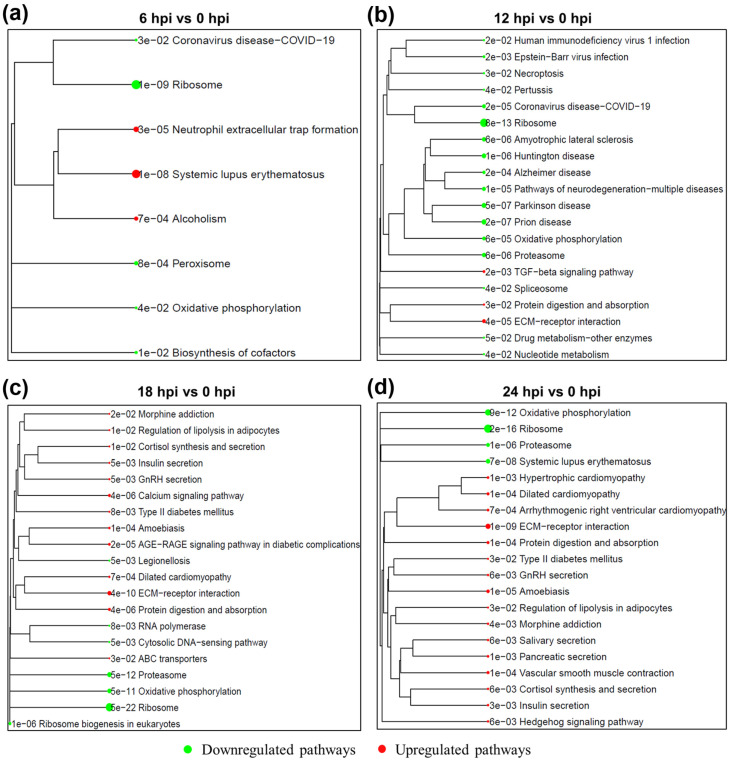
KEGG pathway enrichment analysis of differentially expressed genes at multiple time points following Chandipura virus infection. Dendrograms show clustering of significantly enriched KEGG pathways based on differentially expressed genes (DEGs) at 6 hpi vs. 0 hpi (**a**), 12 hpi vs. 0 hpi (**b**), 18 hpi vs. 0 hpi (**c**), and 24 hpi vs. 0 hpi (**d**). Pathways are color-coded based on enrichment direction: green nodes represent significantly downregulated pathways, and red nodes represent significantly upregulated pathways (adjusted *p*-value < 0.05).

At 6 hpi, upregulated genes were enriched in innate immune response pathways, including response to biotic stimulus and leukocyte activation (Figure 4a). KEGG analysis further revealed enrichment of neutrophil extracellular trap (NET) formation and systemic lupus erythematosus (Figure 5a), indicating early immune activation. Concurrently, ribosome biogenesis, oxidative phosphorylation, and peroxisome pathways were downregulated. At 12 hpi, biological processes related to immune regulation, neuroactive signaling, and cell communication were upregulated, while developmental and differentiation processes were repressed (Figure 4b). KEGG pathways related to chemokine signaling were enriched, whereas neurodegenerative pathways were deregulated (Figure 5b). At 18 hpi, lipid metabolism, inflammatory signaling, and immune regulation pathways were significantly upregulated, while processes related to signal transduction and vesicle-mediated transport were downregulated (Figure 4c). KEGG enrichment included regulation of lipolysis, insulin and cortisol secretion, and calcium signaling, as well as suppression of oxidative phosphorylation, ribosome, and proteasome pathways (Figure 5c). At 24 hpi, a strong downregulation of chromatin organization, RNA processing, and cell cycle pathways was observed (Figure 4d), along with continued KEGG suppression of core metabolic processes (Figure 5d). Meanwhile, enriched pathways in immune dysfunction and hormonal signaling (e.g., systemic lupus erythematosus, GnRH, and cortisol signaling) reflected systemic stress and cellular collapse.

### 2.6. Identification of Drug Candidates Through In Silico Analysis

**Figure 6:**
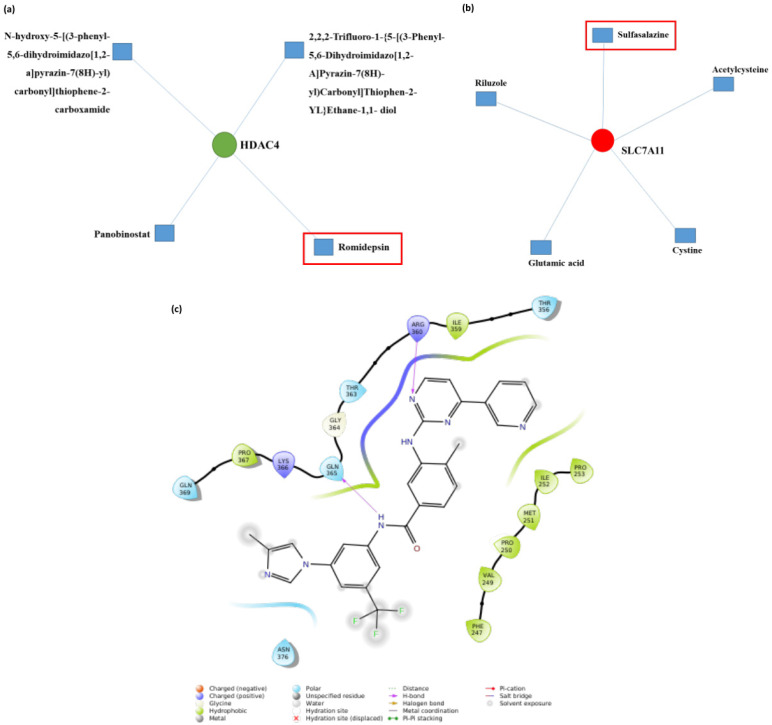
In silico identification of potential drug candidates. Drug–gene interaction network generated using the Network Analyst tool for (**a**) SLC7A11 and (**b**) HDAC4. The target gene is shown as a green node, while interacting drugs/metabolites are shown as blue nodes. Sulfasalazine and romidepsin (highlighted with a red box) were selected based on published literature. (**c**) Predicted protein–ligand interaction map of Nilotinib within the binding pocket of the EGFR1 during the induced fir docking, illustrating key interacting residues and non-covalent interactions that support the network-based prediction.

Based on the gene expression profile at different time points, significantly upregulated genes were identified. Three genes, viz. SLC7A11 (solute carrier family 7 member 11), HDAC4 (histone deacetylase 4), and EGR1 (early growth response 1), were selected based on significant difference in their Log2Fold change and *p*-values (Table 1). In silico protein drug interactions were analyzed through the Network Analyst tool. For SLC7A11 gene, different drug hits were generated in the network analyst, such as sulfasalazine, riluzole, acetylcysteine, glutamic acid, and cysteine (Figure 6a). For HDAC4 gene, drugs such as romidepsin, panobinostat, and other compounds were found to interact (Figure 6b). Among them, sulfasalazine and romidepsin were selected through a literature review. For EGFR1, the Alphafold-predicted protein was used for virtual screening against a library of FDA-approved drugs, which shortlisted potential compounds. In the second stage, induced-fit docking revealed that nilotinib exhibited a docking score of −8.463 and an induced-fit docking score of −722.506, with a prime energy score of −14,279.72. Hydrogen bond interactions were observed with glutamine 365 and arginine 360. The key residues forming the interacting pocket included amino acids ranging from threonine 356 to glutamine 369, phenylalanine 247 to proline 253, as well as asparagine 376 (Figure 6c). Overall, these interactions indicate a stable and well-defined binding of nilotinib within the EGFR1 active pocket, supporting its potential to be an effective drug for EGFR1.

### 2.7. Determination of CHPV Infection Dilution in SHSY5Y Cells

For in vitro drug screening in the relevant human neuroblastoma cell line—SHSY5Y, first the appropriate viral dilution was determined so that cells could survive for at least 48 h, to be able to study the effect of drug. Different dilutions of the CHPV virus lysate were prepared and used for infecting SHSY5Y cells. It was observed that, in 10^−6^ dilution, all the cells were floating within 24 h and at 10^−10^ dilution, and the infection was not well established until 48 h. However, in 10^−8^ dilution, the desired cytopathic effect was observed with some cells still attached to the surface and others in floating condition (Figure S3). Therefore, 10^−8^ dilution was considered as an infection dose for further studies.

### 2.8. In Vitro Drug Screening and Determination of Antiviral Activity

**Figure 7:**
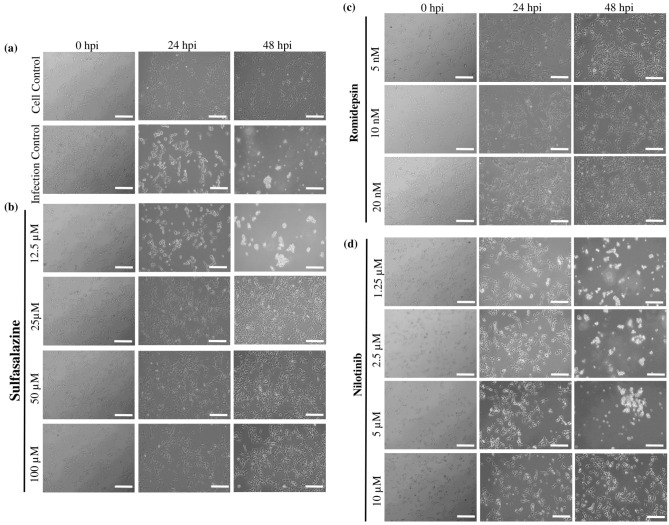
Microscopic images of in vitro analysis of Sulfasalazine, Romidepsin, and Nilotinib drugs in SHSY5Y cells against CHPV infection. (**a**) The top row shows the uninfected control cells and the bottom row shows the CHPV-infected cells at 0 hpi, 24 hpi, and 48 hpi. (**b**) Microscopic images of SHSY5Y cells, treated with different concentrations (12.5 µM, 25 µM, 50 µM, and 100 µM) of Sulfasalazine drug at 0, 24, and 48 h after CHPV infection. (**c**) Microscopic images of cells treated with Romidepsin drug at 5 nM, 10 nM, and 20 nM at 0, 24, and 48 hpi. (**d**) Microscopic images of cells treated with Nilotinib drug at 1.25 µM, 2.5 µM, 5 µM, and 10 µM concentrations. Scale bar: 200 µm.

**Figure 8:**
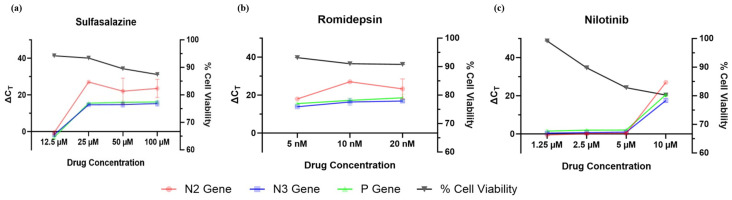
Antiviral activity and cytotoxicity profiling of Sulfasalazine, Romidepsin, and Nilotinib drugs against CHPV. CHPV-infected SHSY5Y cells were treated with increasing concentrations of sulfasalazine (**a**), romidepsin (**b**), or Nilotinib (**c**) and its effect on viral replication was assessed by RT-qPCR. The left *y*-axis shows the ∆C_T_ values for the N2, N3, and P viral genes, where higher ∆C_T_ values indicate reduced viral load. ∆C_T_ was calculated by subtracting C_T_ value of CHPV-infected cells from the C_T_ value of CHPV-infected, drug-treated cells. Cell viability (%), measured at 48 h after drug treatment using the MTT assay, is shown on the right *y*-axis.

Following viral titer determination, selected drug candidates were evaluated in vitro (Figure 7). Sulfasalazine showed dose-dependent antiviral activity: concentrations ≥ 25 µM prevented CHPV-induced cytopathic effects (CPE) at 24 and 48 hpi, whereas 12 µM failed to inhibit CPE (Figure 7b). Romidepsin demonstrated effective antiviral activity at 5–20 nM, with no observable CPE and preserved cell morphology compared to uninfected controls (Figure 7c). Nilotinib exhibited a concentration-dependent response; protection was observed only at 10 µM, while lower concentrations (1.25–5 µM) failed to prevent CPE, which became pronounced by 48 hpi (Figure 7d). Further confirmation was done using RT-qPCR of the viral RNA isolated after 48 h of virus infection and drug treatment (Figure 8). The average C_T_ values for the infection control (without any drug treatment) were found to be 12.96 for N2 gene, 13.99 for N3 gene, and 14.64 for P gene. These C_T_ values were subtracted from the C_T_ values of the drug-treated cells to calculate the ∆C_T_ values. For sulfasalazine, the lowest dose considered for the study was 12.5 µM, which showed no potency against viral infection, whereas the other assessed doses—25 µM, 50 µM, and 100 µM—showed C_T_ values > 30, indicating its efficacy against viral infection even at 25 µM (Figure 8a). In the case of romidepsin, the doses evaluated were 5 nM, 10 nM, and 20 nM and all the doses were found to be effective against CHPV viral infection (Figure 8b). In the case of nilotinib, it was observed that the lower three doses—1.25 µM, 2.5 µM, and 5 µM—showed no major efficacy against CHPV infection; however, the 10 µM dose was found to be effective against CHPV infection (Figure 8c). 

### 2.9. Cytotoxicity Assessment of the Selected Drug Candidates

The cytotoxic effects of sulfasalazine, romidepsin, and Nilotinib were evaluated in SHSY5Y cells using the MTT assay. Cell viability, expressed as a percentage relative to untreated control cells, is depicted by the black line graphs in Figure 8. It was observed that sulfasalazine and romidepsin showed no significant cytotoxic effect on SHSY5Y cells at the concentrations demonstrating antiviral activity, whereas nilotinib showed a significant cytotoxicity beginning at 2.5 µM, which increased in a dose-dependent manner.

## 3. Discussion

Chandipura virus (CHPV) is a neurotropic member of the *Rhabdoviridae* family, known to cause acute encephalitis in young children with a high case fatality rate of 55–75%. The clinical progression of CHPV infection typically begins with non-specific flu-like symptoms and rapidly escalates to severe neurological complications, often resulting in death. Given the short incubation period of approximately 24–48 h, a detailed understanding of the temporal dynamics of host–virus interactions is critical for identifying therapeutic targets that can interfere with viral entry, replication, or immune evasion. Transcriptomic profiling of virus infected host cells provides a powerful approach to identify virus-induced changes in gene expression and thus finding targets that can be utilized for therapeutic intervention. In this context, drug repurposing provides a rapid and practical approach for discovering antiviral candidates by leveraging compounds with known safety and pharmacokinetic profiles. Therefore, investigating the in vitro antiviral efficacy of selected repurposable drugs against CHPV offers a rational approach to identify candidates with the potential for further development.

For transcriptomic analysis and in vitro drug screening study, two distinct cell lines were used. HEK293T cells were chosen for the transcriptomic analysis because of their high permissiveness to viral infection, robust transcriptional activity, and excellent RNA yield and quality, making them widely used in molecular virology, providing a reliable in vitro model to characterize host transcriptional dynamics [16,27,28]. Following gene and drug targets identification, SHSY5Y cells were used for functional drug screening as Chandipura virus is a neurotropic pathogen that primarily affects the central nervous system. SHYSY5Y cells are extensively used as an in vitro neuronal modal to study neurological disorders and neuronal signaling, providing better disease relevance for neurotropic virus infection [29,30,31].

The temporal RNA-seq profiling of CHPV-infected HEK293T cells across 6, 12, 18, and 24 hpi revealed distinct stages of host response—from early innate immune activation to late-stage suppression of core transcriptional machinery. In the absence of extensive literature on CHPV–host interactions, evidence from related rhabdoviruses such as Vesicular Stomatitis Virus (VSV), Rabies virus, and other viruses was utilized to infer potential host responses to CHPV infection.

Initial cytopathic effects were evident as early as 6 hpi, intensifying by 24 hpi, and were accompanied by an increasing number of differentially expressed genes (DEGs), with over 3100 genes showing significant changes across the timeline. Principal Component Analysis and hierarchical clustering confirmed clear temporal segregation of gene expression profiles, suggesting a progressive and highly coordinated host response to CHPV. GO biological process and KEGG pathway enrichment analysis revealed that the response was not static but evolved dramatically with time, moving from immune and signaling activation to metabolic and nuclear function collapse.

At 6 hpi, several early-response genes associated with innate immune sensing and leukocyte activation were upregulated, reflecting rapid host recognition of Chandipura virus (CHPV) infection. IFIT1B, a member of the interferon-stimulated gene family, plays an essential antiviral role by restricting viral RNA translation and replication [32]. GBP3 (guanylate-binding protein 3), another interferon-induced GTPase, is known to modulate inflammasome activation and restrict viral replication by targeting pathogen-containing vacuoles [33]. Upregulation of IL1B indicates early proinflammatory signaling, which is commonly observed during RNA virus infections and contributes to both antiviral defense and inflammation-mediated pathology [34]. The chemokine CCL27, although primarily involved in skin-homing T-cell recruitment, has been reported to participate in immune cell trafficking during viral infections [35], suggesting possible immune cell mobilization in response to CHPV. Interestingly, GPR183 (EBI2), a G-protein–coupled receptor regulating immune cell positioning, was also activated; this receptor has been shown to facilitate immune cell infiltration in viral infections such as influenza and SARS-CoV-2 [36]. The activation of THY1 (CD90) and EGR1, which modulate T-cell activation and early transcriptional responses, further underscores the engagement of immune regulatory pathways. Together, these genes highlight that, within hours of CHPV infection, host cells mount a strong interferon-driven antiviral response coupled with chemokine-mediated immune activation, laying the foundation for the biphasic host response observed at later stages. At 6 hpi, CHPV suppressed several host genes central to immune defense and metabolism, indicating early viral immune evasion. CD83 and BTN3A1/BTN3A2, key costimulatory molecules for T-cell activation, were downregulated, consistent with reports from HIV and HSV where their loss impairs antigen presentation [37,38]. Similarly, repression of IRAK3 and ANXA1 suggests disruption of innate immune signaling and inflammation control, mechanisms exploited by other RNA viruses [39,40]. Several genes, such as LRRK2, TXNIP, and PDK4, were found to be suppressed, which have been previously reported to be upregulated during virus infections [41,42,43]. Collectively, these early transcriptional changes highlight CHPV’s strategy to attenuate host defenses and rewire metabolism to support infection.

At 12 hpi, several host genes linked to immune and signaling pathways were activated, reflecting an intensification of host–virus interactions. CCL27, GPR183 (EBI2), and GBP3 continue to upregulate at 12 hpi too. The upregulation of ERVW-1, an endogenous retroviral envelope gene, is intriguing as ERV elements are known to be transcriptionally activated during viral infections and can influence immune regulation [44]. In addition, angiogenic factors such as VEGFA were also induced, suggesting CHPV infection triggers pathways beyond immunity [45]. Together, these genes highlight an interplay between antiviral defense, immune modulation, and stress adaptation during mid-phase infection. At 12 hpi, alongside the activation of immune and signaling genes, several host genes linked to differentiation and immune regulation were suppressed, suggesting viral strategies to subvert host defenses. IRF4, a transcription factor critical for immune cell differentiation and antiviral responses, was downregulated, which may compromise adaptive immune activation and antigen presentation [46]. Structural and signaling-associated genes such as CNN1 (calponin-1) and TCAP (telethonin), involved in cytoskeletal organization and signaling, were suppressed as well, suggesting disruption of cytoskeletal integrity and cell communication, processes often hijacked by viruses to enhance replication and spread, also reported by other literature [47,48,49]. Collectively, the repression of these genes indicates that while CHPV activates immune signaling pathways, it simultaneously dampens key immune differentiation and regulatory mechanisms, reflecting a dual strategy of activation and suppression to optimize its replication environment.

At 18 hpi, CHPV infection triggered a distinct shift toward immune modulation and metabolic reprogramming. Upregulation of P2RX7, a purinergic receptor, suggests activation of the inflammasome and downstream IL-1β release, processes known to amplify inflammation during viral infections [50]. The induction of AXL, a receptor tyrosine kinase frequently hijacked by viruses such as Zika and Vaccinia, may indicate its role as a cofactor facilitating viral entry or dampening antiviral signaling [51]. Elevated FOXP3 expression points to the activation of regulatory T-cell pathways, potentially contributing to suppression of effector immune responses and favoring viral persistence. In parallel, there was strong enrichment of genes associated with lipid and sphingolipid metabolism, including SPTLC3, FUT2, B3GALT2, and FUT1. These pathways are critical for membrane biosynthesis and remodeling, processes often co-opted by RNA viruses to form replication complexes [52]. Upregulation of sphingolipid biosynthesis, in particular, has been implicated in supporting viral assembly and budding. Collectively, these transcriptional changes at 18 hpi reflect a dual strategy: induction of inflammatory and regulatory immune signals alongside metabolic reprogramming, creating an intracellular environment conducive to CHPV replication. At 18 hpi, several cell-surface receptor signaling and regulatory pathways were suppressed, suggesting that CHPV actively dampens host communication and immune activation. Downregulation of IL18RAP, a critical adaptor for IL-18 receptor signaling, likely interferes with the IL-18-driven activation of NF-κB and IFN-γ production [53], blunting proinflammatory and antiviral responses. Similarly, suppression of FCGR1A (CD64), a high-affinity IgG receptor on monocytes and macrophages, points toward impaired antibody-mediated effector mechanisms and reduced antigen uptake [54]. Additionally, suppression of RSPO2, a positive regulator of Wnt/β-catenin signaling, may interfere with cell proliferation and tissue repair processes during infection [55]. Other suppressed regulators like ANXA1, known for roles in resolution of inflammation [40], and ACSL5, involved in lipid metabolism and apoptosis regulation [56], suggest that CHPV may strategically inhibit both immune responses and metabolic pathways to create a cellular environment favorable for viral persistence.

At 24 hpi, the transcriptional profile was dominated by the activation of chemosensory receptors and solute carrier transporters, reflecting a shift from immune activation toward altered sensory perception and metabolic regulation. A striking feature was the strong upregulation of olfactory receptor genes (OR2AT4, OR10A2, OR10A5, OR2D2, TAS2R3, TAS2R4, PKD1L3). Beyond their role in odor detection, olfactory and taste receptors are increasingly recognized as modulators of immune and inflammatory signaling in non-olfactory tissues [57]. Their induction during viral infection has been linked to stress signaling, cytokine release, and epithelial remodeling, suggesting that CHPV may exploit these receptors to reprogram host signaling pathways. Additionally, solute carrier (SLC) family genes (SLC22A1, SLC6A12, SLC47A2, SLC35F3, SLC7A7) were activated, which are key mediators of amino acid, ion, and drug transport. Viral infections often hijack solute transporters to enhance nutrient availability and maintain ionic balance for replication. However, there are different reports showing up and downregulation of solute transporters in different classes of virus infection [58,59]. The upregulation of KCNMB2/KCNMB3 (potassium channel modulatory subunits) further points to viral modulation of ion flux, which is crucial for viral entry, replication, and virus-induced cytopathic effects [60]. Together, these data suggest that, by 24 hpi, CHPV drives a host state of metabolic exhaustion, sensory signaling disruption, and ion transport remodeling, reflecting advanced manipulation of host pathways to support viral persistence and the onset of cytopathic death. At 24 hpi, CHPV infection led to widespread suppression of genes involved in chromatin regulation, RNA metabolism, and spliceosomal assembly, reflecting a global transcriptional shutdown. Key histone genes (H3C, H4C families) and chromatin regulators such as MED19 and ASF1A were repressed, indicating disruption of nucleosome organization and host transcriptional control, a mechanism commonly exploited by rhabdoviruses to silence host antiviral responses [61]. In parallel, suppression of RNA processing and ribosome assembly genes including NPM3, EIF6, RPS14, GEMIN2, and RRP9 points toward impaired ribosomal maturation and RNA stability [62,63]. Notably, GEMIN2—a core component of the SMN complex essential for spliceosome assembly—was downregulated, alongside spliceosomal proteins such as SNRPB, SNRPG, and SRSF6, suggesting a collapse of host splicing machinery, consistent with viral strategies to favor viral RNA translation over host mRNAs [64,65]. Additionally, repression of RNA modification enzymes (NSUN5, METTL1, TRMT10C) indicates reduced RNA stability and translational efficiency, processes often targeted by RNA viruses to further impair host defense [66]. Together, these findings highlight that by 24 hpi, CHPV imposes a profound blockade on host transcription, RNA processing, and protein synthesis, ensuring host shutoff and prioritization of viral replication.

Based on the significantly deregulated gene expression data, three host targets—SLC7A11, HDAC4 and EGR1—were selected for therapeutic exploration. These genes were selected based on their consistent temporal dysregulation, function relevance to viral replication or host defense, and druggability inferred from pathway and network analyses.

The SLC7A11 gene encodes the xCT subunit of the cystine–glutamate antiporter (system Xc^−^), which is essential for cystine uptake and intracellular glutathione synthesis, thereby regulating redox homeostasis and protection against oxidative stress. Virus-induced oxidative stress is a well-recognized driver of RNA virus replication and host cell damage, making SLC7A11 a functionally relevant antiviral target. Differential regulation of SLC7A11 has been reported during infections with several RNA viruses, implicating it in virus–host metabolic remodeling [67,68,69]. In silico screening identified sulfasalazine, riluzole, N-acetylcysteine, cysteine, and glutamic acid as potential modulators of SLC7A11. Among these, sulfasalazine was prioritized because it directly inhibits system Xc^−^ by blocking cystine transport [70,71], unlike N-acetylcysteine and cysteine, which bypass xCT by replenishing intracellular thiols, or riluzole, which indirectly modulates xCT via glutamatergic signaling. This direct mechanism is particularly relevant in the context of the oxidative stress and lipid metabolic pathways activated during CHPV infection. Additionally, sulfasalazine’s FDA approval and established use in inflammatory and oncological settings supported its selection for antiviral evaluation [72,73,74].

Another prioritized target was HDAC4, a class IIa histone deacetylase involved in chromatin remodeling, transcriptional repression, and immune regulation. Transcriptomic suppression of chromatin organization, RNA processing, and transcriptional machinery at later stages of CHPV infection underscored the relevance of epigenetic regulators as therapeutic targets. Among candidate HDAC inhibitors, romidepsin was selected due to its high potency and documented ability to modulate virus-induced transcriptional programs. Although romidepsin is primarily classified as a class I HDAC inhibitor, several studies report functional activity across multiple HDAC classes at low concentrations, and pan-HDAC effects have been observed in specific cellular contexts. Importantly, we observed a measurable antiviral effect against CHPV in vitro, supporting its functional relevance despite class specificity considerations. Romidepsin’s clinical approval, low effective concentrations, and prior use in studying antiviral, neurological, and immunomodulatory pathways further justified its inclusion [75,76,77,78]. Nonetheless, future studies are required to delineate whether its antiviral activity is mediated through direct HDAC4 modulation or broader epigenetic effects.

The third selected target, EGR1, is an immediate-early transcription factor with context-dependent roles in viral infections, functioning either as an antiviral regulator or as a facilitator of viral replication depending on the virus and cellular environment [79,80,81,82]. Given its early induction during CHPV infection and known regulatory influence on immune and stress-response genes, EGR1 was considered a plausible therapeutic target. Nilotinib, an FDA-approved tyrosine kinase inhibitor, was identified through docking-based analysis as a potential modulator of EGR1-associated signaling. Although nilotinib is primarily used as an anti-cancer drug, it has been previously reported to have antiviral activity against SARS-CoV-2 [83]. While RT-qPCR data indicated partial antiviral activity at higher concentrations, nilotinib also induced dose-dependent cytotoxicity in neuronal SHSY5Y cells, limiting its therapeutic window. In contrast, sulfasalazine and romidepsin demonstrated antiviral activity with minimal cytotoxicity, supporting their prioritization over nilotinib for further development.

## 4. Materials and Methods

### 4.1. Virus and Cells

For culture of Chandipura virus, serum of a 12-year-old male patient was used, which had a low C_T_ value in qPCR. The patient showed symptoms such as high-grade fever, vomiting, and convulsions, and ultimately succumbed to death. HEK293T (human embryonic kidney) and SHSY5Y (human neuroblastoma) cell lines were procured from the National Centre for Cell Science (NCCS), Pune, India. All three cells were maintained in DMEM growth media with 1× antibiotic-antimycotic solution (100 units/mL penicillin, 100 units/mL streptomycin, and 0.25 μg/mL amphotericin B) (HiMedia, Mumbai, India) at 37 °C and 5% CO_2_. HEK293T cells media was supplemented with 10% FBS, whereas SHSY5Y cells were maintained in 20% FBS.

### 4.2. Adaptation of CHPV in HEK293T Cells

The serum was filtered through a 0.22 µm syringe filter and used for infection in HEK293T cells growing in DMEM media supplemented with 2% FBS. Once the cytopathic effect was observed, the cell lysate containing CHPV virus was harvested. After three cycles of freeze-thaw at −80 °C/37 °C, CHPV was obtained, aliquoted, and stored at −80 °C. The virus was well adapted after a few passages and was further used for this study.

### 4.3. Infection of HEK293T Cells with CHPV

First, different dilutions of CHPV lysate were used to infect HEK293T cells to identify the appropriate dilution in which cells survive at least till 48 h so that time-resolved changes can be observed. For infection, cells were seeded in DMEM containing 2.5% FBS in T12.5 cm^2^ flasks. After 12 h, when cells reached around 70% confluency, they were infected with CHPV in 1 mL media. The remaining volume of media was supplemented after 1 h. Cells were incubated at 37 °C and 5% CO_2_. For transcriptomic analysis, cells were collected at 0, 6, 12, 18, and 24 h after infection (hpi). The uninfected cells were used as a control. The experiment was performed in triplicate.

### 4.4. RNA Isolation, Library Construction, and Its Sequencing

The RNA was isolated from all 15 samples (five groups) collected at different time points using the RNeasy Plus Mini Kit (Qiagen, Hilden, Germany) according to the manufacturer’s instructions. RNA purity (OD 260/280) and concentration were determined by Cytation 5 (Biotek, Winooski, VT, USA) and Qubit fluorometer, respectively. RNA integrity was checked using RNase-free agarose gel electrophoresis. The rRNA was depleted using Ribo-zero plus rRNA depletion kit (Illumina, San Diego, CA, USA). After the cleanup, the first strand cDNA synthesis was carried out using random hexamers and ProtoScript II reverse transcriptase provided in the TrueSeq stranded total RNA kit (Illumina). After double-stranded cDNA synthesis, the purified cDNA was ligated using indexing adapters, as per the manufacturer’s protocol, and amplified by PCR to obtain the final paired-end library. The quality and fragment size of the prepared library were confirmed using a LabChip nucleic acid analyzer (Perkin Elmer, Waltham, MA, USA). The prepared library was pooled and sequenced in Novaseq 6000 (Illumina) using 2 × 150 bp read length chemistry in SP flow cell.

### 4.5. Reference Genome Mapping and Transcriptome Assembly

The obtained raw binary base call (BCL) files were converted to FASTQ files using Illumina DRAGEN (Dynamic Read Analysis for GENomics) software, v4.3 (-bcl-conversion-only) [84]. The quality of the obtained reads was determined using the FastQC tool [85]. To ensure high-quality sequencing data, the obtained reads were trimmed using the Fastp tool (version 0.20.0) for adaptor removal, low-quality reads containing more than 10% of unknown nucleotides, and reads with a Q-value ≤ 20 bases [86]. The obtained high-quality reads were mapped to the reference human genome GRCh38.p14 (GenBank assembly accession: GCF_000001405.29) using the STAR alignment tool [87]. The FeatureCounts tool was used to count the abundance of mapped reads in the samples [88].

### 4.6. Differential Expression Analysis, Gene Ontology, and KEGG Pathway Enrichment

The obtained transcripts were further subjected to principal component analysis (PCA) using the R package in R statistical software (version 4.5.0) [89]. For gene expression analysis, RSEM (RNA-seq by Expectation-Maximization) was calculated to quantify transcripts’ abundance and variations. Differential gene expression analysis was performed using the DESeq2 package from Bioconductor (version 3.21) with thresholds of *p*-value < 0.05 and log2 FoldChange ± 1 to identify significantly differentially expressed genes (DEGs) [90]. Gene expression counts were normalized using the variance stabilizing transformation (VST). The most variable genes across samples were selected based on row-wise variance, and expression values were mean-centered per gene to visualize relative expression changes across time points in a heatmap. Bigwig files were generated from BAM files using the RPKM normalization method implemented in bamCoverage from the deepTools package (v3.5.6) [91]. The gene coverage plots were visualized in Integrative Genomics Viewer (v2.18.2) [92]. For gene set enrichment and ontology analysis of significant DEGs, Cluster profiler was used with a *p*-value cut-off < 0.05; pAdjust method—Benjamini and Hochberg and statistical method—Fisher’s exact test [93]. For KEGG pathway analysis, the iDEP (integrated Differential Expression & Pathway analysis) tool (version 2.01) was used with an FDR ratio < 0.05 [94].

### 4.7. In Silico Identification of Potential Drug Candidates

Based on the significant genes identified in the transcriptomic analysis, in silico analysis was performed to identify potential drug candidates that are already FDA-approved and can be repurposed for Chandipura virus infection. The NetworkAnalyst 3.0 tool was utilized to identify antagonists against two significantly upregulated SLC7A11 and HDAC4 genes [95]. From the top candidates, one drug of each gene was selected for in vitro validation based on the literature review. For another significant gene, EGR1, the protein structure was prepared using AlphaFold [96]. The protein was imported into Maestro and prepared for molecular docking. A library of FDA-approved drugs was retrieved from an online source (last accessed on 19 December 2025) [97]. Docking was performed in two stages. In the first stage, Glide high-throughput virtual screening (HTVS) was used to shortlist potential compounds. This was followed by induced-fit docking of the selected candidates. Default parameters were used for both the virtual screening and induced-fit docking steps. The top-ranked compound was subsequently selected for in vitro validation.

### 4.8. In Vitro Testing of Drug Candidates in the SHSY5Y Cell Line

Initially, different dilutions of CHPV lysate were used to infect SHSY5Y cells to identify the appropriate dilution in which cells survive at least 48 h after infection. The SHSY5Y cells were seeded in T12.5 flasks in DMEM supplemented with 20% FBS and incubated for 24 h. The next day, the media was removed and the cells were infected with the CHPV lysate in 10^−6^, 10^−8^, and 10^−10^ dilutions prepared in DMEM with 10% FBS. After 1 h of incubation, the media was replaced with fresh DMEM supplemented with 10% FBS and incubated till 48 h. All the incubations were carried out at 37 °C in a humidified atmosphere of 5% CO_2_ and were observed for cytopathic effect.

Once the viral titer was determined, the in vitro screening of drug candidates was performed. From the in silico analysis and literature review, three drugs, viz. Sulfasalazine (Cat. No. S0883, Sigma-Aldrich, St. Louis, MO, USA), romidepsin (Cat. No. SML1175, Sigma-Aldrich), and nilotinib (Nilocap 200, MSN Laboratories, Hyderabad, India), were selected for in vitro screening. The concentrations of drugs were determined based on the previously published literature of in vitro testing [83,98,99,100,101,102,103]. For sulfasalazine, 12.5 µM, 25 µM, 50 µM and 100 µM doses; for romidepsin, 5 nM, 10 nM, and 20 nM doses; and for nilotinib, 1.25 µM, 2.5 µM, 5 µM, and 10 µM were selected. SHYSY5Y cells were given infection as mentioned above with the pre-determined dilution of CHPV. After 1 h, the media was replaced with different concentrations of drugs dissolved in DMEM supplemented with 10% FBS were overlaid and incubated for 48 h. All the incubations were carried out at 37 °C in a humidified atmosphere of 5% CO_2_. Once the incubation was completed, cells were harvested and proceeded further for RT-PCR. All the experiments were performed in duplicates and independently repeated twice.

### 4.9. Extraction of Viral RNA and Real-Time RT-PCR

Viral RNA from the harvested cells was isolated using QIAamp viral RNA kit (Cat. No. 52904, Qiagen). Multiplex RT-qPCR was performed targeting three genes (N2, N3, and P) of CHPV to determine the viral load after drug treatment. The RT-qPCR reaction mixture (12.5 µL) consisted of 3 µL of Taqpath 1-step mastermix, 445 nM forward primer, 445 nM reverse primer, and 115 nM probe of each gene, 2.75 µL nuclease free water, and 3 µL RNA template.

N2 Gene: Forward Primer: TCGTAGCTGCTGTGGATATGT

Reverse Primer: AACCCAGGTGAAGACCTCCT

Probe: TGTCCACTCTGTCGCACCTCCA

N3 Gene: Forward Primer: GATTTGTTGCGGATGATGAC

Reverse Primer: CCAGAAATGGAAACTGGGAT

Probe: TCGACATGGGCTTGTCCACGA

P Gene: Forward Primer: TTTAATCGACATGGGAGCAATTG

Reverse Primer: TAAGGTGGGTCAGACGGAGAGA

Probe: AGAATTCATCCTGGCAGCT

The RT-qPCR was performed in a 7500 real-time PCR system (Applied Biosystems, Foster City, CA, USA). The thermal cycling conditions of RT-qPCR consisted of holding step for 2 min at 25 °C, for 10 min at 53 °C, for 2 min at 95 °C, and 40 cycles of denaturation for 15 min at 95 °C and for 30 s at 60 °C. Further, the viral load was determined by calculating ∆C_T_ and % inhibition compared to infected cells without drug treatment.

### 4.10. Determination of In Vitro Cytotoxicity of Drug Candidates

The cytotoxicity of used drug candidates on SHSY5Y cells was determined by 3-(4,5-dimethylthiazol-2-yl)-2,5-diphenyltetrazolium bromide (MTT) Assay. A total of 20,000 cells per well were seeded in a 96-well plate in DMEM supplemented with 20% FBS and incubated. After 24 h, the media was aspirated and the cells were treated with the drug candidates of different concentrations such as sulfasalazine (12.5 µM, 25 µM, 50 µM, 100 µM), romidepsin (5 nM, 10 nM, 20 nM) and nilotinib (1.25 µM, 2.5 µM, 5 µM, 10 µM) and incubated for 48 h. All the incubation procedures were conducted at 37 °C in a humidified atmosphere of 5% CO_2_. After the treatment period, 5 mg/mL of MTT reagent was added to the well and incubated for 4 h in the dark at 37 °C. After 4 h, the media was carefully withdrawn without disturbing the formazan crystals and further DMSO was added to the well and mixed gently until the crystals dissolved. Further, the absorbance was read at 570 nm in a multimode plate reader (Cytation 5, Biotek).

## 5. Conclusions

In conclusion, this study provides one of the first comprehensive molecular characterizations of host responses to Chandipura virus, a pathogen that remains significantly underexplored despite its high neurovirulence and outbreak potential. By integrating time-resolved transcriptomic profiling with in silico target prioritization and in vitro drug validation, this study represents the first report to systematically link dynamic host transcriptional remodeling during CHPV infection with functional evaluation of repurposable antiviral candidates. The identification of sulfasalazine and romidepsin as host-directed inhibitors with favorable antiviral and cytotoxicity profiles underscores the translational potential of this approach. Given the limited availability of targeted therapeutics and the scarcity of mechanistic studies on CHPV, these findings establish a foundational framework for future antiviral development and mechanistic exploration. As transcriptomic resources for emerging and neglected viruses continue to expand, this integrated strategy offers a scalable blueprint for accelerating therapeutic discovery against poorly studied neurotropic viral pathogens.

## Figures and Tables

**Table 1:** Fold changes of selected genes during different time points.

Gene	6 h	12 h	18 h	24 h	*p*-Value	Adjusted *p*-Value
SLC7A11	1.56	6.59	3.18	2.16	1.26 × 10^−18^	1.82 × 10^−14^
HDAC4	2.71	1.83	2.23	2.07	3.78 × 10^−07^	4.78 × 10^−6^
EGR1	13.64	−0.94	5.17	2.50	3.15 × 10^−10^	2.36 × 10^−8^

## Data Availability

The raw RNA-sequencing datasets generated and analyzed during this study have been deposited in the Indian Biological Data Centre (IBDC) and are accessible under INDC accession number PRJEB109108. Processed data and the list of differentially expressed genes are provided in the Supplementary Materials.

## Supplementary Materials

The following supporting information can be downloaded at: https://www.mdpi.com/article/10.3390/ijms27083364/s1.

## References

[1] Bhatt P.N., Rodrigues F.M. (1967). Chandipura: A new Arbovirus isolated in India from patients with febrile illness. Indian J. Med. Res..

[2] Kemp G.E. (1975). Viruses other than arenaviruses from West African wild mammals. Factors affecting transmission to man and domestic animals. Bull. World Health Organ..

[3] Peiris J., Dittus W., Ratnayake C. (1993). Seroepidemiology of dengue and other arboviruses in a natural population of toque macaques (*Macaca sinica*) at Polonnaruwa, Sri Lanka. J. Med. Primatol..

[4] Rao B., Basu A., Wairagkar N.S., Gore M.M., Arankalle V.A., Thakare J.P., Jadi R.S., Rao K., Mishra A. (2004). A large outbreak of acute encephalitis with high fatality rate in children in Andhra Pradesh, India, in 2003, associated with Chandipura virus. Lancet.

[5] Adnan H.M., Salomon I.M., Ahmed M.M. (2025). Chandipura virus outbreak: A growing public health crisis that demands immediate action. Ann. Med. Surg..

[6] (2024). Acute Encephalitis Syndrome Due to Chandipura Virus-India. Disease Outbreak News [Internet]. http://www.who.int/emergencies/disease-outbreak-news/item/2024-DON529.

[7] Aderao G.N., Nikhil K.C., Sarkar S., Patel S.K., Kanaka K.K., Mhaske V.S., Kumar A., Bin Emran T. (2026). Emergence of Chandipura viral encephalitis in India: A strategic approach to combat a fatal viral epidemic. Ann. Med. Surg..

[8] Menghani S., Chikhale R., Raval A., Wadibhasme P., Khedekar P. (2012). Chandipura Virus: An emerging tropical pathogen. Acta Trop..

[9] Jadi R.S., Sudeep A.B., Kumar S., Arankalle V.A., Mishra A.C. (2010). Chandipura virus growth kinetics in vertebrate cell lines, insect cell lines & embryonated eggs. Indian J. Med. Res..

[10] Sapkal G.N., Sawant P.M., Mourya D.T. (2018). Chandipura Viral Encephalitis: A Brief Review. Open Virol. J..

[11] Sudeep A., Gurav Y., Bondre V. (2016). Changing clinical scenario in Chandipura virus infection. Indian J. Med. Res..

[12] Basak S., Mondal A., Polley S., Mukhopadhyay S., Chattopadhyay D. (2007). Reviewing Chandipura: A vesiculovirus in human epidemics. Biosci. Rep..

[13] Truong T.T.T., Liu Z.S.J., Panizzutti B., Kim J.H., Dean O.M., Berk M., Walder K. (2024). Network-based drug repurposing for schizophrenia. Neuropsychopharmacology.

[14] Wyler E., Mösbauer K., Franke V., Diag A., Gottula L.T., Arsiè R., Klironomos F., Koppstein D., Hönzke K., Ayoub S. (2021). Transcriptomic profiling of SARS-CoV-2 infected human cell lines identifies HSP90 as target for COVID-19 therapy. iScience.

[15] Nyayanit D.A., Sarkale P., Baradkar S., Patil S., Yadav P.D., Shete-Aich A., Kalele K., Gawande P., Majumdar T., Jain R. (2020). Transcriptome & viral growth analysis of SARS-CoV-2-infected Vero CCL-81 cells. Indian J. Med. Res..

[16] Sun G., Cui Q., Garcia G., Wang C., Zhang M., Arumugaswami V., Riggs A.D., Shi Y. (2021). Comparative transcriptomic analysis of SARS-CoV-2 infected cell model systems reveals differential innate immune responses. Sci. Rep..

[17] Cao Y., Zhang K., Liu L., Li W., Zhu B., Zhang S., Xu P., Liu W., Li J. (2019). Global transcriptome analysis of H5N1 influenza virus-infected human cells. Hereditas.

[18] Wang X.P., Wen B., Zhang X.J., Ma L., Liang X.L., Zhang M.L. (2022). Transcriptome Analysis of Genes Responding to Infection of Leghorn Male Hepatocellular Cells with Fowl Adenovirus Serotype 4. Front. Vet. Sci..

[19] Yang R., Yan H., Wang Y., Yang W., Qin J. (2025). Transcriptome Analysis Reveals Gemykibivirus Infection Induces Mitochondrial DNA Release in HEK293T Cells. Viruses.

[20] Yi H., Yang S., Deng J., Liu X., Zhang X., Xie X., Li J., Wang J., Wei L., Zheng Z. (2026). Cross-Cell line transcriptome profiling reveals host-Virus interactions in monkeypox virus infection. Emerg. Microbes Infect..

[21] Arriaga-Canon C., Contreras-Espinosa L., Rebollar-Vega R., Montiel-Manríquez R., Cedro-Tanda A., García-Gordillo J.A., Álvarez-Gómez R.M., Jiménez-Trejo F., Castro-Hernández C., Herrera L.A. (2022). Transcriptomics and RNA-Based Therapeutics as Potential Approaches to Manage SARS-CoV-2 Infection. Int. J. Mol. Sci..

[22] Salgado-Albarrán M., Navarro-Delgado E.I., Del Moral-Morales A., Alcaraz N., Baumbach J., González-Barrios R., Soto-Reyes E. (2021). Comparative transcriptome analysis reveals key epigenetic targets in SARS-CoV-2 infection. npj Syst. Biol. Appl..

[23] Niarakis A., Ostaszewski M., Mazein A., Kuperstein I., Kutmon M., Gillespie M.E., Funahashi A., Acencio M.L., Hemedan A., Aichem M. (2023). Drug-target identification in COVID-19 disease mechanisms using computational systems biology approaches. Front. Immunol..

[24] Rajput A., Thakur A., Rastogi A., Choudhury S., Kumar M. (2021). Computational identification of repurposed drugs against viruses causing epidemics and pandemics via drug-target network analysis. Comput. Biol. Med..

[25] Dezső Z., Ceccarelli M. (2020). Machine learning prediction of oncology drug targets based on protein and network properties. BMC Bioinform..

[26] Zhang X., Wu F., Yang N., Zhan X., Liao J., Mai S., Huang Z. (2022). In silico Methods for Identification of Potential Therapeutic Targets. Interdiscip. Sci..

[27] Chen H., Charles P.D., Gu Q., Liberatori S., Robertson D.L., Palmarini M., Wilson S.J., Mohammed S., Castello A. (2025). Omics Analyses Uncover Host Networks Defining Virus-Permissive and -Hostile Cellular States. Mol. Cell. Proteom..

[28] Li M., Xiong J., Zhou H., Liu J., Wang C., Jia M., Wang Y., Zhang N., Chen Y., Zhong T. (2025). Transcriptomic and Proteomic Analysis of Monkeypox Virus A5L-Expressing HEK293T Cells. Int. J. Mol. Sci..

[29] Zanker J., Hüser D., Savy A., Lázaro-Petri S., Hammer E.-M., Schwarzer C., Heilbronn R. (2023). Evaluation of the SH-SY5Y cell line as an in vitro model for potency testing of a neuropeptide-expressing AAV vector. Front. Mol. Neurosci..

[30] Mignolet M., Gilloteaux J., Halloin N., Gueibe M., Willemart K., De Swert K., Bielarz V., Suain V., Pastushenko I., Gillet N.A. (2023). Viral Entry Inhibitors Protect against SARS-CoV-2-Induced Neurite Shortening in Differentiated SH-SY5Y Cells. Viruses.

[31] Ferdous J., Makino H., Masatani T., Fujimoto Y., Naitou K., Shiraishi M. (2025). In vitro susceptibility of differentiated SH-SY5Y human neuroblastoma cells to herpes simplex virus type 1 and Japanese encephalitis virus infection. J. Vet. Med. Sci..

[32] Fensterl V., Sen G.C. (2015). Interferon-induced Ifit proteins: Their role in viral pathogenesis. J. Virol..

[33] Tretina K., Park E.-S., Maminska A., MacMicking J.D. (2019). Interferon-induced guanylate-binding proteins: Guardians of host defense in health and disease. J. Exp. Med..

[34] Bawazeer A.O., Rosli S., Harpur C.M., Docherty C.A., Mansell A., Tate M.D. (2021). Interleukin-1β exacerbates disease and is a potential therapeutic target to reduce pulmonary inflammation during severe influenza A virus infection. Immunol. Cell Biol..

[35] Homey B., Alenius H., Müller A., Soto H., Bowman E.P., Yuan W., McEvoy L., Lauerma A.I., Assmann T., Bünemann E. (2002). CCL27-CCR10 interactions regulate T cell-mediated skin inflammation. Nat. Med..

[36] Foo C.X., Bartlett S., Chew K.Y., Ngo M.D., Bielefeldt-Ohmann H., Arachchige B.J., Matthews B., Reed S., Wang R., Smith C. (2023). GPR183 antagonism reduces macrophage infiltration in influenza and SARS-CoV-2 infection. Eur. Respir. J..

[37] Hock B.D., Kato M., McKenzie J.L., Hart D.N.J. (2001). A soluble form of CD83 is released from activated dendritic cells and B lymphocytes, and is detectable in normal human sera. Int. Immunol..

[38] Herrmann T., Karunakaran M.M. (2022). Butyrophilins: γδ T Cell Receptor Ligands, Immunomodulators and More. Front. Immunol..

[39] Mahmoud I.S., Jarrar Y.B., Febrimarsa (2023). Modulation of IRAK enzymes as a therapeutic strategy against SARS-CoV-2 induced cytokine storm. Clin. Exp. Med..

[40] Resende F., de Araújo S., Tavares L.P., Teixeira M.M., Costa V.V. (2023). The Multifaceted Role of Annexin A1 in Viral Infections. Cells.

[41] Herbst S., Gutierrez M.G. (2019). LRRK2 in Infection: Friend or Foe?. ACS Infect. Dis..

[42] Pan M., Zhang F., Qu K., Liu C., Zhang J. (2022). TXNIP: A Double-Edged Sword in Disease and Therapeutic Outlook. Oxid. Med. Cell. Longev..

[43] Kido H. (2015). Influenza virus pathogenicity regulated by host cellular proteases, cytokines and metabolites, and its therapeutic options. Proc. Jpn. Acad. Ser. B Phys. Biol. Sci..

[44] Bao C., Gao Q., Xiang H., Shen Y., Chen Q., Gao Q., Cao Y., Zhang M., He W., Mao L. (2024). Human endogenous retroviruses and exogenous viral infections. Front. Cell. Infect. Microbiol..

[45] Alkharsah K.R. (2018). VEGF Upregulation in Viral Infections and Its Possible Therapeutic Implications. Int. J. Mol. Sci..

[46] Forero A., Moore P.S., Sarkar S.N. (2013). Role of IRF4 in IFN-stimulated gene induction and maintenance of Kaposi sarcoma-associated herpesvirus latency in primary effusion lymphoma cells. J. Immunol..

[47] Walsh D., Naghavi M.H. (2019). Exploitation of Cytoskeletal Networks during Early Viral Infection. Trends Microbiol..

[48] Taylor M.P., Koyuncu O.O., Enquist L.W. (2011). Subversion of the actin cytoskeleton during viral infection. Nat. Rev. Microbiol..

[49] De Conto F., Mancabelli L., Prandini E., Ventura M. (2025). Highly Dynamic Cytoskeletal Networks Support Productive Viral Infection and Host Innate Immune Response Activation. Curr. Clin. Microbiol. Rep..

[50] Di Virgilio F., Dal Ben D., Sarti A.C., Giuliani A.L., Falzoni S. (2017). The P2X7 Receptor in Infection and Inflammation. Immunity.

[51] Meertens L., Labeau A., Dejarnac O., Cipriani S., Sinigaglia L., Bonnet-Madin L., Le Charpentier T., Hafirassou M.L., Zamborlini A., Cao-Lormeau V.-M. (2017). Axl Mediates ZIKA Virus Entry in Human Glial Cells and Modulates Innate Immune Responses. Cell Rep..

[52] Mazzon M., Mercer J. (2014). Lipid interactions during virus entry and infection. Cell. Microbiol..

[53] Hedl M., Zheng S., Abraham C. (2014). The IL18RAP region disease polymorphism decreases IL-18RAP/IL-18R1/IL-1R1 expression and signaling through innate receptor-initiated pathways. J. Immunol..

[54] Zhang H., Li L., Liu L. (2018). FcγRI (CD64) contributes to the severity of immune inflammation through regulating NF-κB/NLRP3 inflammasome pathway. Life Sci..

[55] Srivastava A., Rikhari D., Srivastava S. (2024). RSPO2 as Wnt signaling enabler: Important roles in cancer development and therapeutic opportunities. Genes Dis..

[56] Luo Q., Das A., Oldoni F., Wu P., Wang J., Luo F., Fang Z. (2023). Role of ACSL5 in fatty acid metabolism. Heliyon.

[57] Orecchioni M., Matsunami H., Ley K. (2022). Olfactory receptors in macrophages and inflammation. Front. Immunol..

[58] Moskovskich A., Goldmann U., Kartnig F., Lindinger S., Konecka J., Fiume G., Girardi E., Superti-Furga G. (2019). The transporters SLC35A1 and SLC30A1 play opposite roles in cell survival upon VSV virus infection. Sci. Rep..

[59] Nguyen N.N.T., Lim Y.-S., Nguyen L.P., Tran S.C., Luong T.T.D., Nguyen T.T.T., Pham H.T., Mai H.N., Choi J.-W., Han S.-S. (2018). Hepatitis C Virus Modulates Solute carrier family 3 member 2 for Viral Propagation. Sci. Rep..

[60] Hover S., King B., Hall B., Loundras E.-A., Taqi H., Daly J., Dallas M., Peers C., Schnettler E., McKimmie C. (2016). Modulation of Potassium Channels Inhibits Bunyavirus Infection. J. Biol. Chem..

[61] Herbein G., Wendling D. (2010). Histone deacetylases in viral infections. Clin. Epigenet..

[62] Wang X., Zhu J., Zhang D., Liu G. (2022). Ribosomal control in RNA virus-infected cells. Front. Microbiol..

[63] Dong H.-J., Zhang R., Kuang Y., Wang X.-J. (2021). Selective regulation in ribosome biogenesis and protein production for efficient viral translation. Arch. Microbiol..

[64] Sehrawat S., Garcia-Blanco M.A. (2023). RNA virus infections and their effect on host alternative splicing. Antivir. Res..

[65] Li R., Gao S., Chen H., Zhang X., Yang X., Zhao J., Wang Z. (2023). Virus usurps alternative splicing to clear the decks for infection. Virol. J..

[66] Zou Y., Guo Z., Ge X.-Y., Qiu Y. (2024). RNA Modifications in Pathogenic Viruses: Existence, Mechanism, and Impacts. Microorganisms.

[67] Banerjee S., Sarkar R., Mukherjee A., Mitra S., Gope A., Chawla-Sarkar M. (2024). Rotavirus-induced lncRNA SLC7A11-AS1 promotes ferroptosis by targeting cystine/glutamate antiporter xCT (SLC7A11) to facilitate virus infection. Virus Res..

[68] Rabinowitz J., Sharifi H.J., Martin H., Marchese A., Robek M., Shi B., Mongin A.A., de Noronha C.M. (2021). xCT/SLC7A11 antiporter function inhibits HIV-1 infection. Virology.

[69] Liu Y., Tang H., Xu P., Zhou X., Li S. (2025). SARS-CoV-2 N protein interacts with SLC7A11 to cause ferroptosis in acute lung injury. Allergol. Immunopathol..

[70] Gout P.W., Buckley A.R., Simms C.R., Bruchovsky N. (2001). Sulfasalazine, a potent suppressor of lymphoma growth by inhibition of the x(c)-cystine transporter: A new action for an old drug. Leukemia.

[71] Dixon S.J., Patel D.N., Welsch M., Skouta R., Lee E.D., Hayano M., Thomas A.G., Gleason C.E., Tatonetti N.P., Slusher B.S. (2014). Pharmacological inhibition of cystine–glutamate exchange induces endoplasmic reticulum stress and ferroptosis. eLife.

[72] Zhao C., Yu Y., Yin G., Xu C., Wang J., Wang L., Zhao G., Ni S., Zhang H., Zhou B. (2024). Sulfasalazine promotes ferroptosis through AKT-ERK1/2 and P53-SLC7A11 in rheumatoid arthritis. Inflammopharmacology.

[73] Yang J., Zhou Y., Xie S., Wang J., Li Z., Chen L., Mao M., Chen C., Huang A., Chen Y. (2021). Metformin induces Ferroptosis by inhibiting UFMylation of SLC7A11 in breast cancer. J. Exp. Clin. Cancer Res..

[74] Hu K., Li K., Lv J., Feng J., Chen J., Wu H., Cheng F., Jiang W., Wang J., Pei H. (2020). Suppression of the SLC7A11/glutathione axis causes synthetic lethality in KRAS-mutant lung adenocarcinoma. J. Clin. Investig..

[75] Falkenberg K.J., Johnstone R.W. (2014). Histone deacetylases and their inhibitors in cancer, neurological diseases and immune disorders. Nat. Rev. Drug Discov..

[76] Saha S., Pal D. (2023). Role of Histone Deacetylase Inhibitors on Viral Replication: A Review. Biointerface Res. Appl. Chem..

[77] Feng Q., Su Z., Song S., Xu H., Zhang B., Yi L., Tian M., Wang H. (2016). Histone deacetylase inhibitors suppress RSV infection and alleviate virus-induced airway inflammation. Int. J. Mol. Med..

[78] Jønsson K.L., Tolstrup M., Vad-Nielsen J., Kjær K., Laustsen A., Andersen M.N., Rasmussen T.A., Søgaard O.S., Østergaard L., Denton P.W. (2015). Histone Deacetylase Inhibitor Romidepsin Inhibits De Novo HIV-1 Infections. Antimicrob. Agents Chemother..

[79] Woodson C.M., Kehn-Hall K. (2022). Examining the role of EGR1 during viral infections. Front. Microbiol..

[80] Zhao Y., Sui L., Wu P., Li L., Liu L., Ma B., Wang W., Chi H., Wang Z.-D., Wei Z. (2023). EGR1 functions as a new host restriction factor for SARS-CoV-2 to inhibit virus replication through the E3 ubiquitin ligase MARCH8. J. Virol..

[81] Zhu Z., Du X., Li P., Zhang X., Yang F., Cao W., Tian H., Zhang K., Liu X., Zheng H. (2018). Early Growth Response Gene-1 Suppresses Foot-and-Mouth Disease Virus Replication by Enhancing Type I Interferon Pathway Signal Transduction. Front. Microbiol..

[82] Wong L.M., Li D., Tang Y., Méndez-Lagares G., Thompson G.R., Hartigan-O’cOnnor D.J., Dandekar S., Jiang G. (2022). Human Immunodeficiency Virus-1 Latency Reversal via the Induction of Early Growth Response Protein 1 to Bypass Protein Kinase C Agonist-Associated Immune Activation. Front. Microbiol..

[83] Cagno V., Magliocco G., Tapparel C., Daali Y. (2021). The tyrosine kinase inhibitor nilotinib inhibits SARS-CoV-2 in vitro. Basic Clin. Pharmacol. Toxicol..

[84] Illumina DRAGEN Secondary Analysis. Version 4.3 ed2024. https://www.illumina.com/products/by-type/informatics-products/dragen-secondary-analysis.html.

[85] Andrews S. (2010). FastQC: A Quality Control Tool for High Throughput Sequence Data. https://www.bioinformatics.babraham.ac.uk/projects/fastqc/.

[86] Chen S. (2023). Ultrafast one-pass FASTQ data preprocessing, quality control, and deduplication using fastp. iMeta.

[87] Dobin A., Davis C.A., Schlesinger F., Drenkow J., Zaleski C., Jha S., Batut P., Chaisson M., Gingeras T.R. (2012). STAR: Ultrafast universal RNA-seq aligner. Bioinformatics.

[88] Liao Y., Smyth G.K., Shi W. (2013). feature Counts: An efficient general purpose program for assigning sequence reads to genomic features. Bioinformatics.

[89] Camargo A. (2022). PCAtest: Testing the statistical significance of Principal Component Analysis in R. PeerJ.

[90] Love M.I., Huber W., Anders S. (2014). Moderated estimation of fold change and dispersion for RNA-seq data with DESeq2. Genome Biol..

[91] Ramírez F., Ryan D.P., Grüning B., Bhardwaj V., Kilpert F., Richter A.S., Heyne S., Dündar F., Manke T. (2016). deepTools2: A next generation web server for deep-sequencing data analysis. Nucleic Acids Res..

[92] Robinson J.T., Thorvaldsdóttir H., Winckler W., Guttman M., Lander E.S., Getz G., Mesirov J.P. (2011). Integrative genomics viewer. Nat. Biotechnol..

[93] Wu T., Hu E., Xu S., Chen M., Guo P., Dai Z., Feng T., Zhou L., Tang W., Zhan L. (2021). clusterProfiler 4.0: A universal enrichment tool for interpreting omics data. Innovation.

[94] Ge S.X., Son E.W., Yao R. (2018). iDEP: An integrated web application for differential expression and pathway analysis of RNA-Seq data. BMC Bioinform..

[95] Zhou G., Soufan O., Ewald J., Hancock R.E.W., Basu N., Xia J. (2019). NetworkAnalyst 3.0: A visual analytics platform for comprehensive gene expression profiling and meta-analysis. Nucleic Acids Res..

[96] Jumper J., Evans R., Pritzel A., Green T., Figurnov M., Ronneberger O., Tunyasuvunakool K., Bates R., Žídek A., Potapenko A. (2021). Highly accurate protein structure prediction with AlphaFold. Nature.

[97] https://file.selleckchem.com/downloads/library/20220301-L1300-FDA-approved-Drug-Library.SDF.

[98] Sehm T., Fan Z., Ghoochani A., Rauh M., Engelhorn T., Minakaki G., Dörfler A., Klucken J., Buchfelder M., Eyüpoglu I.Y. (2016). Sulfasalazine impacts on ferroptotic cell death and alleviates the tumor microenvironment and glioma-induced brain edema. Oncotarget.

[99] Zou L., Gao Z., Zeng F., Xiao J., Chen J., Feng X., Chen D., Fang Y., Cui J., Liu Y. (2019). Sulfasalazine suppresses thyroid cancer cell proliferation and metastasis through T-cell originated protein kinase. Oncol. Lett..

[100] Cosenza M., Civallero M., Fiorcari S., Pozzi S., Marcheselli L., Bari A., Ferri P., Sacchi S. (2016). The histone deacetylase inhibitor romidepsin synergizes with lenalidomide and enhances tumor cell death in T-cell lymphoma cell lines. Cancer Biol. Ther..

[101] Radhakrishnan V., Song Y.-S., Thiruvengadam D. (2008). Romidepsin (depsipeptide) induced cell cycle arrest, apoptosis and histone hyperacetylation in lung carcinoma cells (A549) are associated with increase in p21 and hypophosphorylated retinoblastoma proteins expression. Biomed. Pharmacother..

[102] Wu J., Xu X., Zheng L., Mo J., Jin X., Bao Y. (2021). Nilotinib inhibits microglia-mediated neuroinflammation to protect against dopaminergic neuronal death in Parkinson’s disease models. Int. Immunopharmacol..

[103] Dong H., Wen C., He L., Zhang J., Xiang N., Liang L., Hu L., Li W., Liu J., Shi M. (2024). Nilotinib boosts the efficacy of anti-PDL1 therapy in colorectal cancer by restoring the expression of MHC-I. J. Transl. Med..

