# Supplementary material for: Time-Resolved Transcriptomic Profiling of Chandipura Virus Infection Reveals Dynamic Host Responses and Host-Directed Therapeutic Targets
Source: Int J Mol Sci. 2026 Apr 9;27(8):3364. doi: 10.3390/ijms27083364 (PMC13115980; doi:10.3390/ijms27083364)
Supplement: Supplementary file 1 [file ijms-27-03364-s001.zip › Supplementary Figures.pdf]

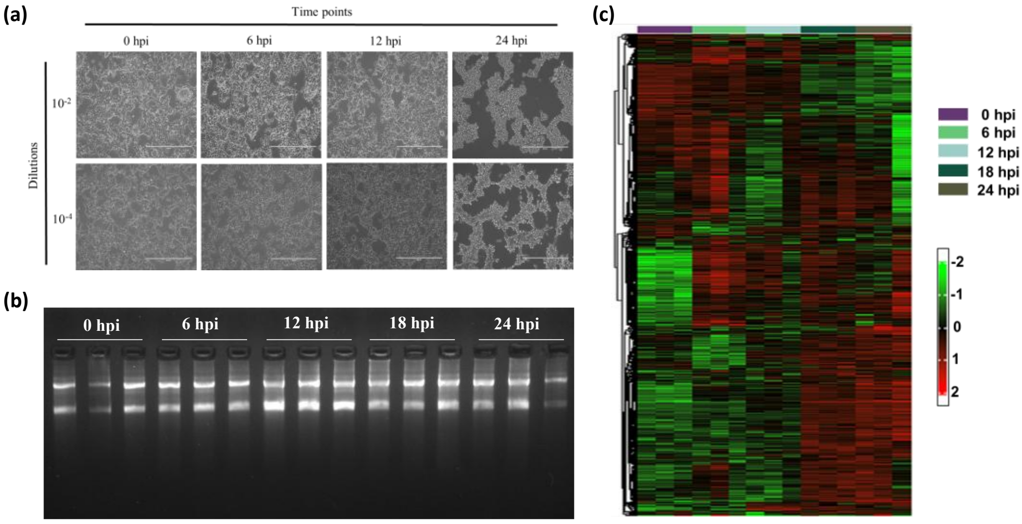

Figure S1

Figure S1. (a) Microscopic images of HEK293T cells infected with Chandipura virus at varying dilutions and time points. Bright-field images showing cytopathic effects in HEK293T cells infected with different dilutions (10^{-2} and 10^{-4}) of Chandipura virus at 0, 6, 12, and 24 hpi. Progressive cytopathic effects are visible with increasing time and viral concentration. Scale bar: 400 µm. **(b)** Agarose gel electrophoresis of total RNA extracted from HEK293T cells at different time points post Chandipura virus infection**.** Gel image shows distinct and sharp 28S and 18S ribosomal RNA bands at all-time points, indicating high-quality and intact RNA. (c) Hierarchical clustering heatmap of significant differentially expressed genes across all time points. Rows represent genes, and columns represent samples (in triplicates). Red indicates upregulation and green indicates downregulation relative to control.

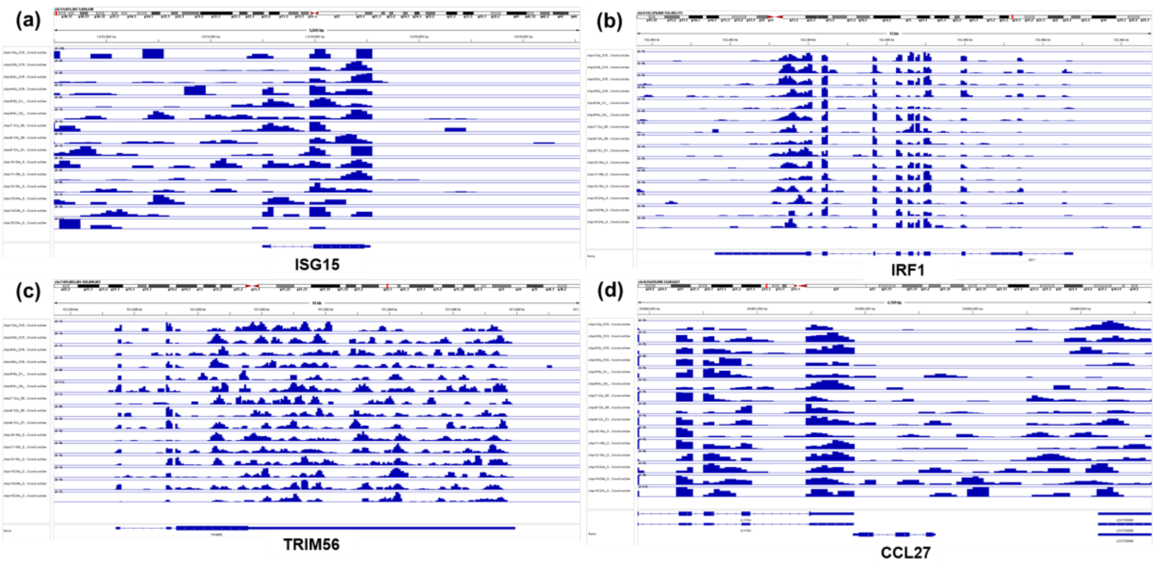

Figure S2

Figure S2. Genome browser visualization of RNA-seq coverage across representative immune response genes during CHPV infection. Normalized RNA-seq coverage tracks across the loci of (a) ISG15, (b) IRF1, (c) TRIM56, and (d) CCL27 during CHPV infection. Tracks correspond to samples collected at 0, 6, 12, 18, and 24 hpi, with three biological replicates per time point. The histograms represent normalized read coverage across each genomic locus, illustrating temporal variation in transcriptional signal during infection.

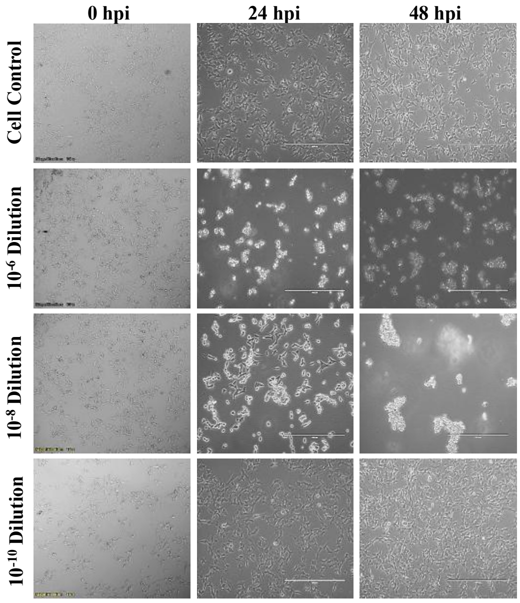

Figure S3

Figure S3. Microscopic images of SHSY5Y cells infected with different dilutions of Chandipura virus. Representative bright-field microscopic images of SHSY5Y cells with 10^{-6}, 10^{-8}, 10^{-10} viral dilutions at different time points (0, 24 and 48 hpi).
