# Supplementary material for: Time-Resolved Transcriptomic Profiling of Chandipura Virus Infection Reveals Dynamic Host Responses and Host-Directed Therapeutic Targets
Source: Int J Mol Sci. 2026 Apr 9;27(8):3364. doi: 10.3390/ijms27083364 (PMC13115980; doi:10.3390/ijms27083364)
Supplement: Supplementary file 1 [file ijms-27-03364-s001.zip › Supplementary Tables.pdf]

Supplementary Tables

Table S1: Statistics of the RNA-seq dataset

Sample Name	Raw Reads (millions)	Clean Reads (millions)	Raw Bases (millions)	Clean Bases (millions)	Q20 (%)	Q30 (%)	GC (%)	Mapped reads (%)
0 hpi (A)	40.45	39.51	5875.43	5539.61	98.77	96.15	47.7	85.00
0 hpi (B)	42.21	41.49	6166.05	5858.02	98.81	96.22	47.2	85.75
0 hpi (C)	40.97	39.93	5930.78	5719.72	98.92	96.64	47.4	85.46
6 hpi (A)	38.21	37.67	5525.95	5255.87	98.82	96.18	47.6	86.76
6 hpi (B)	35.07	34.41	5138.45	4866.61	98.7	95.97	47.4	87.10
6 hpi (C)	52.03	51.45	7550.96	7203.31	98.7	95.9	46.99	86.41
12 hpi (A)	34.19	33.32	4974.54	4676.47	98.66	95.84	46.07	85.62
12 hpi (B)	39.36	38.52	5664.21	5344.62	98.68	95.89	45.25	86.79
12 hpi (C)	43.20	42.48	6253.16	5932.54	98.73	96.01	47.37	83.86
18 hpi (A)	29.08	28.58	4241.91	4022.73	98.69	95.92	46.24	86.99
18 hpi (B)	45.86	45.16	6652.86	6320.54	98.71	95.96	46.78	86.24
18 hpi (C)	38.05	36.16	5463.67	5002.93	98.52	95.65	47.34	86.50
24 hpi (A)	40.57	39.27	5740.19	5350.05	98.71	96.09	47.42	80.94
24 hpi (B)	50.96	49.76	7230.43	6828.59	98.75	96.09	47.62	80.70
24 hpi (C)	51.69	50.43	7097.29	6658.80	98.73	96.08	44.01	75.24
**Total**	621.9	608.21	89505.88	84580.41				
**Average**	41.46	40.54	5967.06	5638.69	98.73	96.04	46.83	84.62

Table S2: List of biological processes and their related activated and suppressed genes

**6 hpi**	
**Activated genes**	
Biological process involved in interspecies interaction between organisms	MIR421, IFIT1B, GBP3, CCL27, TRIM54, IL1B
Leukocyte activation	THY1, CLCF1, EGR1, IL1B, TBC1D10C, GPR183, CPLX2
Lymphocyte activation	THY1, CLCF1, EGR1, IL1B, TBC1D10C, GPR183
Response to biotic stimulus	MIR421, IFIT1B, GBP3, CCL27, TRIM54, IL1B
Response to external biotic stimulus	MIR421, IFIT1B, GBP3, CCL27, TRIM54, IL1B
Response to other organism	MIR421, IFIT1B, GBP3, CCL27, TRIM54, IL1B

**Suppressed genes**	
Angiogenesis	ANXA1, PGF, RLN2, EFNA1, CALCRL, APOLD1, APLN, TNFAIP2, PDGFRA, MINAR2
Cell surface receptor protein tyrosine kinase signaling pathway	FUZ, ADAMTS3, EFNB3, CAV2, PGF, STON1, EFNA1, TXNIP, PAK3, AFAP1L2, CD8A, PDK4, APLN, SAMD10, MTSS1, PDGFRA
Cell-cell adhesion via plasma membrane adhesion molecules	AMIGO1, PCDH1, PCDH18, NECTIN4, CLDN1, DSC1
Cilium movement	BBS4, CFAP68, BBS2, TTC12, IQCG, SPA17, DNAI4, AKAP3, PFN4, ENKUR
Flagellated sperm motility	BBS4, CFAP68, BBS2, TTC12, IQCG, AKAP3, PFN4, ENKUR
G protein-coupled receptor signaling pathway	RGS2, CAV2, ANXA1, ARRDC3, CALCRL, LRRK2, APLN, PTAFR, GPR158, OR51B5
Interleukin-6 production	AFAP1L2, PTAFR, IRAK3, TMEM106A
Locomotory behaviour	ARRDC3, ALDH1A3, LRRK2, DMBX1, SELENOP, MINAR2
Positive regulation of cytokine production	CD83, ANXA1, BTN3A1, BTN3A2, AFAP1L2, RBM47, LRRK2, PTAFR, TRIM6, IRAK3, TMEM106A
Regulation of exocytosis	CDK5, RAB3D, PFN2, SDC1, C9orf72, RAB3C, STXBP6, ANXA1, SLC4A8, LRRK2, PTAFR
Regulation of interleukin-6 production	AFAP1L2, PTAFR, IRAK3, TMEM106A
Regulation of tumor necrosis factor production	LRRK2, PTAFR, IRAK3, TMEM106A
**12 hpi**	
**Activated genes**	
Anatomical structure morphogenesis	KRT35, NR2E3, ERVW-1, CNTN4, ADM2, HOXD12, FUT1, ERVFRD-1, VLDLR
Interspecies interaction between organisms	MIR421, GBP3, CCL27
Cell communication	MIR421, ADA2,
Cellular response to stimulus	MIR421, GBP3, ADA2, CCL27, NR2E3, MEIOB, CHGB, CHAC1, NPY6R, ADM2, INHBE, OR6A2, SLC7A11, OR10A2, GPR183
Defense response	GBP3, CCL27
G protein-coupled receptor signaling pathway	ADM2, OR6A2, OR10A2, GPR183, TAS2R3, GNG3, GPRASP1, ACKR4, TAS2R4, GPR135, GPR50, GPR21, OPRD1,
Immune response	GBP3, CCL27
Immune system process	GBP3, CCL27, SLC7A11, GPR183
Nervous system process	NR2E3, KCNMB3, OR6A2, SLC7A11, OR10A2, TAS2R3, VLDLR
Organelle organization	KRT35, MEIOB, ODAD1, NEAT1
Positive regulation of signal transduction	CTH, LRRK1, DDR2, DKK2, CYP1B1, BMPER, G0S2, CITED1, VEGFA, NOTCH2NLA
Regulation of cell population proliferation	ZBED3, CTH, LRRK1, DDR2, DKK2, CYP1B1, BMPER, G0S2, CITED1, VEGFA, NOTCH2NLA
Response to biotic stimulus	NR2E3, NEAT1, SLC7A11, GPR183, FUT1
Response to chemical	MIR421, GBP3, CCL27
Response to external biotic stimulus	MIR421
Response to external stimulus	MIR421, GBP3, CCL27
Response to other organism	MIR421

Small molecule metabolic process	MIR421, GBP3, CCL27
System process	ADA2, SPTLC3, SLC7A11, FUT1, VLDLR, CYP3A5, DDIT4, ASNS
**Suppressed genes**	
Cell differentiation	IL4I1, TCAP, PGLYRP1, IRF4
Cellular developmental process	IL4I1, TCAP, PGLYRP1, IRF4
Multicellular organismal process	IL4I1, TCAP, PGLYRP1, CNN1, MYBPHL, IRF4
Regulation of multicellular organismal process	IL4I1, PGLYRP1, CNN1, IRF4
**18 h**	
**Activated genes**	
Defense response to bacterium	GBP3, P2RX7, DEFB131B
Detection of chemical stimulus	OR10A5, KCNMB3, OR10A2, OR6A2, TAS2R3, TAS2R4
Leukocyte homeostasis	P2RX7, AXL, FOXP3, GPR183
Membrane lipid biosynthetic process	P2RX7, SPTLC3, FUT2, ZNF750, B3GALT2, FUT1
Membrane lipid metabolic process	P2RX7, SPTLC3,
Regulation of production of molecular mediator of immune response	P2RX7, AXL, FOXP3, CLCF1, HPX
Sphingolipid biosynthetic process	P2RX7, SPTLC3, FUT2, ZNF750, B3GALT2, FUT1
Sphingolipid metabolic process	P2RX7, SPTLC3, FUT2, ZNF750, B3GALT2, FUT1
**Suppressed genes**	
Cell surface receptor signaling pathway	ACSL5, P2RX3, IL18RAP, RSPO2, FCGR1A
Establishment of localization in cell	YIPF7, P2RX3, OAZ3, TERB1, ABRA
Positive regulation of response to stimulus	NTS, P2RX3, IL18RAP, RSPO2, ABRA, FCGR1A
Positive regulation of signal transduction	NTS, P2RX3, RSPO2, ABRA
Positive regulation of transport	ANXA1, ACSL5, P2RX3, OAZ3, FCGR1A
Regulation of signal transduction	NTS, LYNX1, ACSL5, P2RX3, RSPO2, ABRA
**24 h**	
**Activated genes**	
Detection of chemical stimulus	OR2AT4, OR10A5, OR2D2, KCNMB3, OR10A2, TAS2R3, KCNMB2, TAS2R4, PKD1L3
Detection of stimulus	OR2AT4, OR10A5, NR2E3, OR2D2, KCNMB3, OPN1SW, OR10A2, TAS2R3, KCNMB2, TAS2R4, PKD1L3
Detection of stimulus involved in sensory perception	OR2AT4, OR10A5, OR2D2, OR10A2, TAS2R3, TAS2R4, PKD1L3, CEP250, EYS
Organic cation transport	SLC25A2, SLC22A1, SLC6A12, SLC47A2, SLC35F3, SLC7A7
Sensory perception of chemical stimulus	OR2AT4, OR10A5, OR2D2, OR10A2, TAS2R3, TAS2R4, PKD1L3
Detection of stimulus involved in sensory perception	OR2AT4, OR10A5, OR2D2, KCNMB3, OR10A2, TAS2R3, KCNMB2, TAS2R4, PKD1L3
**Suppressed genes**	
Protein-DNA complex organization	MED19, H3C2, GTF2A2, GTF2B, GRWD1, H4C4, TAF7, PSMC6, GMNN, POLE3, H3C6, TBP, H4C8, H4C3, MED8, ASF1A, CENPV, MIS12, H3C7, H3C10, H1-3, CENPX, H3C4, H3C1, H4C16, BRF2, H3C12, H2BC11, H4C14, H4C2, H4C11, MED31, H3C13, TAF11L12
Regulation of ERK1 and ERK2 cascade	SPRY4, PDGFD

Regulatory ncRNA-mediated post-transcriptional gene silencing	MIR7-1, MIR186, MIRLET7D, MIR616
RNA processing	NPM3, DEDD2, AAR2, GEMIN7, C1D, SF3B5, RPL27, CTU2, MRTO4, FASTKD5, NSUN5, EIF6, RBM11, TADA1, RRP7BP, TOE1, SNRNP25, SNORA71A, LAGE3, SNHG7, SNORA16A, TRMU, SNORD45C, TU1, FDXACB1, RPS14, RRP9, METTL18, DUS3L, TRMT1, INTS5, RNF113A, PPIL1, SNRPG, TSSC4, RPS21, DIMT1, TXNL4B, DDX39A, FASTKD2, SNORA32, CLP1, GEMIN2, PUSL1, TFB2M, POP7, PRKRA, METTL1, SNORA3B, SNHG1, SNORA12, SRSF7, BCDIN3D, SNORD32A, TRMT10C, PHF5A, SNORD35A, SRSF6, TAF12-DT, RSRP1, THOC6, TRMT61A, FASTKD3, ABT1, SNORD76, RNVU1-15, TARBP2, LCMT2, SCARNA2, SNORA65, YRDC, SNORA72, SNORD35B, EMG1, SNRPB, SNORD12B, SNORD79, SNORD83A, SNORD50B, SNORD22, SNORA66, IMP3, RNVU1-6, RPP25L, RNVU1-4, SNORD21, SNORA26, SNORD47, SNORA5C, LSM10, SNORD38B, SCARNA12, SNORD44, SNORD139, SNORD80, SNORD14A, SNORD110, SNORA13, SNORD20, SNORA27, SNORD18B, SNORD58A, RNVU1-7, SNORD3D, SNORD46, SNORD3B-1, SNORD28, RNVU1-8, SNORA19, SNORD27, SNORA1, SNORD18C, SNORA25, SNORD78, SNORD31, SNORD45B, ADAT3, SNORD74, SNORD23, SNORD6, SNORD99, SNORD100, SNORD83B, SNORD37, SNORD19, SNORD12, SNORA30, SNORD5, SNORD14D, SNORD12C, SNORD77, SNORD3C
Spliceosomal complex assembly	SNRPG, GEMIN2, PHF5A, SRSF6, TAF12-DT, RSRP1, RNVU1-15, SNRPB, RNVU1-6, RNVU1-4, RNVU1-7, RNVU1-8
